# Risk-Factors for Soft-Tissue Injuries, Lacerations and Fractures During Racing in Greyhounds in New Zealand

**DOI:** 10.3389/fvets.2021.737146

**Published:** 2021-12-03

**Authors:** Anna L. Palmer, Chris W. Rogers, Kevin J. Stafford, Arnon Gal, Charlotte F. Bolwell

**Affiliations:** ^1^School of Veterinary Science, Massey University, Palmerston North, New Zealand; ^2^School of Agriculture and Environment, Massey University, Palmerston North, New Zealand; ^3^Department of Veterinary Clinical Medicine, College of Veterinary Medicine, University of Illinois at Urbana-Champaign, Urbana, IL, United States

**Keywords:** greyhound, greyhound racing, injury, risk factor, canine sport

## Abstract

Recognition of injuries in racing animals is essential to identify potential risk factors so actions can be taken to reduce or mitigate the cause of the injury to safeguard the animal. Racing greyhounds are subject to musculoskeletal injuries associated with athletic pursuit, in particular soft-tissue injuries, lacerations, and fractures. The objective of this study was therefore to determine risk factors for soft-tissue injuries, lacerations and fractures occurring during racing, using a cohort of greyhounds racing in New Zealand between 10th September 2014 and 31st July 2020. Dog-level, race-level and track-level risk factors for each outcome were assessed using mixed-effects multivariable logistic regression including trainer as a random effect. Throughout the study period there were 218,700 race starts by 4,914 greyhounds, with a total of 4,385 injuries. Of these, 3,067 (69.94%) were classed as soft-tissue injuries, 641 (14.62%) were reported as lacerations, and 458 (10.44%) were fractures. Greyhounds with a low racing frequency (racing more than 7 days apart) had 1.33 [95% confidence interval (CI): 1.06–1.67] times the odds of fracture compared to those racing more frequently. Older greyhounds had a greater odds of fracture compared with younger greyhounds. Racing every 7 days had a lower odds of soft-tissue injury compared with racing more than once a week. Dogs over 39 months had 1.53 (95% CI: 1.35–1.73) times the odds of sustaining a soft-tissue injury compared to the younger dogs. Greyhounds originating from Australia had a higher odds of fracture and laceration compared with New Zealand dogs. Better performing dogs (higher class) had a greater odds of fracture and laceration whilst maiden dogs had a higher odds of soft-tissue injury. Greyhounds starting from the outside box had a higher odds of fracture. There was considerable variation in the odds of soft-tissue injury at different racetracks. In conclusion, although the incidence of soft-tissue injuries was higher than other injury types, the repercussion of such injuries was less than those for fractures. The results from this study will help to inform intervention strategies aimed at reducing the rate of injuries in racing greyhounds, enhancing racing safety and greyhound welfare.

## Introduction

Within New Zealand there is an increasing quantity of data published from which it is possible to describe the greyhound (*Canis familiaris*) racing population and the structure of racing. This baseline data provides the opportunity to identify areas requiring improvements in order to safeguard the welfare of racing greyhounds. The current industry structure provides greyhounds with a constant opportunity to race throughout the year (1). These greyhounds race a median of every seven days and begin their racing career at a median of 21 months of age (1). In New Zealand, greyhounds race in an eight-dog field, around a circular sand track, chasing and finishing on a mechanical artificial lure. At the end of the race the full field of dogs congregate around the lure while it slows and comes to a stop before they are caught by their handler. Not only do the greyhounds show a periodicity to the pattern of racing (1), but a previous study has reported that regardless of the number of times a greyhound races in a week, they have two high-intensity workload sessions, either training or racing, each week (2). Workloads, including training and competition load, contribute to injuries through fitness or fatigue. The physiological adaptations associated with each of these, as well as exposure to extrinsic (external) risk factors and the injury mechanism, wherein the biomechanical stress of an event is greater than the tolerance of the athlete, results in an injury occurring (3, 4).

Many factors influence the risk of injuries in racing greyhounds and studies have reported dog- and race- related risk factors, such as weight (5, 6), race age (6, 7), sex (7), speed at which the dogs race (6, 7), track design and surface (6, 8–11), race distance (9), grade of race (9) and environmental-factors such as weather (9) and month of the year (9). Across these studies there is disparity in the risk factors identified and the magnitude of the risk reported that may reflect the governance of greyhound racing of the country in which they were examined. Moreover, there are differences between racing jurisdictions in the type and frequency of racing injuries sustained by racing greyhounds (6, 10, 12). In Australasia, soft-tissue injuries, fractures and lacerations are the most common race-day injuries reported for racing greyhounds (6, 12). Despite this, studies have focused on risk factors for all injury types collectively. It is widely recognized that risk factors for different injury outcomes will vary, and thus there is a need to identify risk factors specific to New Zealand racing for each of the common injury outcomes.

With limited data on injuries in racing greyhounds and obvious differences between racing jurisdictions, there was a need for a New Zealand specific study. Accordingly, the objective of the present study was to investigate risk factors for injuries described as “soft-tissue injuries,” “lacerations,” or “fractures” reported during racing for greyhounds in New Zealand. Identification of risk factors that influence racing injuries will facilitate the necessary improvements and development of strategies that may reduce the incidence of injuries in racing greyhounds.

## Materials and Methods

### Study Design, Population, and Data Collection

The study utilized data from all race starts and all injuries occurring during greyhound races in New Zealand between 10th September 2014 and 31st July 2020. Data were supplied as Microsoft Excel files by Greyhound Racing New Zealand (GRNZ) and included race-day veterinary injury information, race start data and greyhound information data. The study population consisted of all greyhounds declared to race in at least one race during the study period, excluding dogs that were scratched before a race or deemed unfit to race upon a pre-kenneling veterinary examination. To capture all information on racing injuries, a cohort of dogs was created, which included greyhounds that had their first race start on or after the 10^th^ September 2014.

As part of normal race-day procedures, GRNZ-approved on-track veterinarians clinically examined every greyhound due to race that day before it was secured in its pre-race kennel. Any greyhounds deemed unfit to race are not allowed to race. Immediately after each race, Stipendiary Stewards monitor and review the recording of the race and any greyhounds considered to have sustained an injury during or after the race are sent to the on-track veterinarian for a clinical examination. Furthermore, the on-track veterinarian and/or trainer can request a greyhound be checked after it has raced [Rule 56.1—Greyhound Racing New Zealand (13)]. Details of any injuries sustained are recorded on standardized forms (Supplementary Figure 1) and then entered into the GRNZ injury database by the Stipendiary Steward chairing the race meeting; only one (the main) injury per dog per race start was entered into the database. The inclusion criteria for an injury in this study was defined as any event that involved a greyhound requiring veterinary attention on race-day and for which an injury report was generated in the GRNZ database. A greyhound could have several starts and multiple injuries over the study period. A fatality was defined as an injury that resulted in the death or euthanasia of the greyhound on race-day.

Injury data included the anatomical location of injury, the severity of the injury, the place on the track where the injury occurred, and the imposed stand-down period required for the greyhound to recover. Race level data included the race date, box number (randomly allocated position in the starting boxes), finishing position, trainer name, greyhound name, race grade [the grade of the race where class 0 (C0) are maiden races, class 1 (C1) are lower grade races and there is a successive increase to the higher grade races through to class 5 (C5)], meeting location (track) and race distance. Greyhound level information included whelp date (date born), date of qualifying trial, date of first start, sex of greyhound, country in which the greyhound was whelped, microchip number and the greyhound's ear tattoo. Details of the anatomical location (numbered as per a supplied diagram) of the injury, as well as the penetrometer (device used to measure track firmness) reading for the track surface, were only broadly described in the data, which prevented a detailed analysis of these variables.

In this study, injuries were categorized as soft-tissue injuries (general soreness, muscle tear, muscle sprain), lacerations, fractures, or other injuries (split webbing, cramp, hemorrhage, and swelling). The type of and cause of injury re-categorized where descriptions that were not as common in the population were combined to form an “other” category for each variable.

### Statistical Methods

Data were organized for analysis in Microsoft Access 2016 and Microsoft Excel 2016 (Microsoft Corporation, Redmond, WA, USA) and the integrity of the data were checked using exploratory data analysis. Race date and date whelped were used to create new variables including age at the time of the race (race age) and number of days between racing starts. New variables were created for race year, season (spring: September - November, summer: December – February, autumn: March-May, winter: June-August), whether the greyhound was injured during the race and whether the greyhound was fatally injured or died during the race. Injury stand-down period was grouped according to the categories recognized by Greyhound Racing New Zealand where the number of days the greyhound is not allowed to race reflects the severity of the injury. Injuries were separately categorized by severity using the method described by Beer (6), where injuries were grouped into four categories by the number of days the greyhound received as a stand-down: “Minor injuries” (1–7 day stand-down), “Moderate injuries” (8–20 day stand-down), “Serious injuries” (>21 day stand-down), and “Catastrophic injuries” (death or euthanasia). The number of races in different time periods were considered, however, given that most dogs race a median of 7 days (IQR: 3–9), this was collinear with the time-varying variables (number of races in the previous 7-day period, 14-day period, 21-day period, and 28-day period) and so the days since the previous race start, which best described the data, was measured instead. New datasets were created for each injury description (soft-tissue injury, laceration, and fracture) and each dataset contained a binary outcome variable coding for the specific injury description for that dataset.

Normality of continuous data were assessed with the Shapiro-Wilk test. Continuous data that were non-normally distributed were summarized with medians and interquartile range (IQR). Injuries were summarized as counts and percentages by variables describing the type, cause, anatomical location, and severity of injuries.

Mixed effects logistic regression modeling was used to determine explanatory variables that were associated with each of the three outcomes. Collinearity between continuous variables was assessed by calculating pairwise Pearson's correlation coefficients, where if pairs of variables had moderate or high correlation (*p* > 0.4), the variable that best described the data were assessed in the multivariable model. The age at race start and the career race start number were highly correlated, as were the time-varying variables and the number of days since the previous racing start. Age at race start was retained in the models with the outcomes of fracture and soft-tissue injury, while the career race start number was retained in the model with the outcome of laceration. The linearity of continuous variables was assessed, and if non-linearity was identified, the variables were categorized based on quartiles or biologically plausible groupings (age of greyhound, career race start number, and days since previous racing start). The age at racing start variable was categorized into evenly sized groups: 14–25, 26–31, 32–38, and 39–77 months. Career race start number was categorized into groups based on quartiles and the days since previous racing start variable was grouped in accordance to industry relevance (1) (more than one race per week, racing once per week and racing less than once a week). An initial logistic regression model was fitted to each dataset that contained only an intercept term and a random-effect coding for trainer or dog, to determine the presence of clustering at the trainer- or dog-level. The parameter accounting for the most variance was subsequently included in the final models.

Potential explanatory variables were screened using univariable mixed effects logistic regression, and variables were selected for inclusion in subsequent multivariable models if they had a calculated probability of *p* < 0.2. A backwards stepwise model building process was then used to develop a multivariable model and selection of variables was based on the likelihood ratio test *p*-value and/or Akaike information criterion. Variables with a likelihood ratio *p* < 0.05 were retained in the model. Likelihood ratio tests were used to determine the significance of variables in the model and confounding was assessed by examining the effect of addition of variables on parameter estimates (a change of ≥20%). Biologically plausible two-way interaction terms were then considered for inclusion in the multivariable model. Separate analyses were performed for each of the three injury outcomes (soft-tissue injury, laceration, and fracture). The fitted probability of the outcome for each model was calculated based on the final mixed effects logistic regression multivariable model. Residual values were calculated to assess the fit of the model and residuals close to 0 reflected a well-fitting model.

Statistical analyses were conducted in Stata version 15 (College Station, StataCorp LP, TX, USA).

## Results

During the study period there were 218,700 race starts by 4,914 individual greyhounds. There were 4,385 injuries reported, of which 3,067 (69.94%) were classed as soft-tissue injuries, 641 (14.62%) were reported as lacerations, 458 (10.44%) were reported as fractures, and 219 (4.99%) were reported as “other” injuries. The distribution of racing starts and injuries is shown in Table 1. Of the 4,914 individual dogs, 2,602 (52.95%) sustained at least one racing injury. Injury descriptions classified by the severity of the injury in terms of stand-down days are presented in Table 2. The overall incidence of injury was 20.05 per 1,000 starts (95% CI: 19.47–20.65) and 14.02 (95% CI: 13.54–14.53), 2.93 (95% CI: 2.71–3.17) and 2.09 (95% CI: 1.91–2.29) per 1,000 starts for soft-tissue injuries, lacerations and fractures, respectively. There were 235 catastrophic injuries, and the overall incidence of catastrophic injuries was 1.07 per 1,000 starts (95% CI: 0.94–1.22). Approximately half the fracture cases (*n* = 227/458; 49.56%) were associated with a fatal outcome, two (0.31%) lacerations and two (0.07%) of the soft-tissue injuries were associated with a fatal outcome.

**Table 1:** The distribution (number and percentage) of racing starts and injuries that occurred during greyhound races in New Zealand from 10th September 2014 to 31st July 2020.

**Variable**	**n starts (%)**	**n injuries (%)**	**n fractures (%)**	**n lacerations (%)**	**n soft-tissue injuries (%)**
**Sex**					
Dog	126,560 (57.87)	2,531 (57.72)	286 (62.45)	393 (61.31)	1,719 (56.05)
Bitch	92,140 (42.13)	1,854 (42.28)	172 (37.55)	248 (38.69)	1,348 (43.95)
**Country of origin**					
New Zealand	166,923 (76.33)	3,181 (72.54)	307 (67.03)	439 (68.49)	2,287 (74.57)
Australia	51,777 (23.67)	1,204 (27.46)	151 (32.97)	202 (31.51)	780 (25.43)
**Race age (months)**					
14–25	59,548 (27.23)	897 (20.46)	85 (18.56)	139 (21.68)	632 (20.61)
26–31	57,871 (26.46)	1,039 (23.69)	117 (25.55)	158 (24.65)	718 (23.41)
32–38	50,746 (23.20)	1,088 (24.81)	127 (27.73)	146 (22.78)	760 (24.78)
39–77	50,535 (23.11)	1,361 (31.04)	129 (28.17)	198 (30.89)	957 (31.20)
**Career start number**					
1–13 starts	58,051 (26.54)	1,129 (25.75)	117 (25.55)	171 (26.68)	781 (25.46)
14–28 starts	52,386 (23.95)	980 (22.35)	97 (21.18)	148 (23.09)	690 (22.50)
29–51 starts	53,884 (24.64)	1,080 (24.63)	136 (29.69)	142 (22.15)	752 (24.52)
51–231 starts	54,379 (24.86)	1,196 (27.27)	108 (23.58)	180 (28.08)	844 (27.52)
**Days since previous race**					
<7	86,537 (39.57)	1,719 (39.20)	155 (33.84)	269 (41.97)	1,211 (39.48)
7	68,940 (31.52)	1,271 (28.89)	145 (31.66)	190 (29.64)	873 (28.46)
>7	63,223 (28.91)	1,395 (31.81)	158 (34.50)	182 (28.39)	983 (32.05)
**Race type**					
Sprint	142,530 (65.17)	2,945 (67.16)	306 (66.81)	423 (65.99)	2,082 (67.88)
Middle	72,026 (32.93)	1,358 (30.97)	141 (30.79)	200 (31.20)	936 (30.52)
Distance	4,144 (1.89)	82 (1.87)	11 (2.40)	18 (2.81)	49 (1.60)
**Race grade**					
Class 0	34,037 (15.56)	643 (14.66)	52 (11.35)	94 (14.66)	467 (15.23)
Class 1	73,000 (33.38)	1,456 (33.20)	135 (29.48)	195 (30.42)	1,045 (34.07)
Class 2	37,642 (17.21)	819 (18.68)	74 (16.16)	117 (18.25)	591 (19.27)
Class 3	25,845 (11.82)	530 (12.09)	61 (13.32)	85 (13.26)	358 (11.67)
Class 4	18,750 (8.57)	368 (8.39)	49 (10.70)	45 (7.02)	259 (8.44)
Class 5	22,514 (10.29)	457 (10.42)	75 (16.38)	86 (13.42)	272 (8.87)
Other	6,912 (3.16)	112 (2.55)	12 (2.62)	19 (2.96)	75 (2.45)
**Racetrack**					
Track A	66,352 (30.34)	818 (18.65)	145 (31.66)	176 (27.46)	441 (14.38)
Track B	13,009 (5.95)	122 (2.78)	17 (3.71)	28 (4.37)	64 (2.09)
Track C	27,213 (12.44)	686 (15.64)	79 (17.25)	118 (18.41)	466 (15.19)
Track D	52,386 (23.95)	1,508 (34.39)	113 (24.67)	165 (25.74)	1,151 (37.53)
Track E	20,641 (9.44)	347 (7.91)	37 (8.08)	58 (9.05)	229 (7.47)
Track F	13,283 (6.07)	125 (2.85)	21 (4.59)	19 (2.96)	77 (2.51)
Track G	25,816 (11.80)	779 (17.77)	46 (10.04)	77 (12.01)	639 (20.83)
**Starting box**					
1	27,396 (12.53)	527 (12.02)	37 (8.08)	71 (11.08)	393 (12.81)
2	27,408 (12.53)	572 (13.04)	56 (12.23)	75 (11.7)	413 (13.47)
3	27,259 (12.46)	578 (13.18)	62 (13.54)	87 (13.57)	404 (13.17)
4	27,381 (12.52)	580 (13.23)	56 (12.23)	87 (13.57)	401 (13.07)
5	27,213 (12.44)	550 (12.54)	61 (13.32)	82 (12.79)	377 (12.29)
6	27,324 (12.49)	576 (13.14)	58 (12.66)	98 (15.29)	385 (12.55)
7	27,374 (12.52)	504 (11.49)	56 (12.23)	75 (11.70)	351 (11.44)
8	27,345 (12.50)	498 (11.36)	72 (15.72)	66 (10.30)	343 (11.18)
**Season**					
Winter	59,433 (27.18)	1,221 (27.84)	106 (23.14)	171 (26.68)	877 (28.59)
Spring	51,719 (23.65)	1,040 (23.72)	108 (23.58)	168 (26.21)	727 (23.70)
Summer	54,465 (24.90)	1,097 (25.02)	117 (25.55)	146 (22.78)	775 (25.27)
Autumn	53,083 (24.27)	1,027 (23.42)	127 (27.73)	156 (24.34)	688 (22.43)
**Race year**					
2019/2020	40,419 (18.48)	1,044 (23.81)	77 (16.81)	134 (20.90)	773 (25.20)
2018/2019	46,344 (21.19)	1,011 (23.06)	105 (22.93)	129 (20.12)	746 (24.32)
2017/2018	46,137 (21.10)	793 (18.08)	87 (19.00)	124 (19.34)	540 (17.61)
2016/2017	41,769 (19.10)	652 (14.87)	87 (19.00)	105 (16.38)	423 (13.79)
2015/2016	32,419 (14.82)	683 (15.58)	73 (15.94)	109 (17.00)	464 (15.13)
2014/2015	11,612 (5.31)	202 (4.61)	29 (6.33)	40 (6.24)	121 (3.95)

*Data were collected from 218,700 racing starts and included 4,385 injuries*.

**Table 2:** Description of the type of injury by the severity of the injury for injuries occurring during greyhound races in New Zealand from 10th September 2014 to 31st July 2020 (*n* = 4,385).

	**Soft-tissue injury**		**Laceration**		**Fracture**		**Other^a^**		**Total injuries**	
	***n***	**%**	***n***	**%**	***n***	**%**	***n***	**%**	**n**	**%**
Minor injuries^b^	242	7.89	76	11.86	3	0.66	28	12.79	349	7.96
Moderate injuries^c^	1,861	60.68	494	77.07	17	3.71	140	63.93	2,512	57.29
Serious injuries^d^	962	31.37	69	10.76	211	46.07	47	21.46	1,289	29.40
Death/euthanized	2	0.07	2	0.31	227	49.56	4	1.83	235	5.36
Total	3,067		641		458		219		4,385	

a *Other included Split webbing (n = 147/4,385), Cramp (n = 49/4,385), Hemorrhaged (n = 8/4,385) and Swelling (n = 8/4,385)*.

b *Minor injuries includes injuries with a stand-down period of <7 days*.

c *Moderate injuries includes injuries with a stand-down period of 7–20 days*.

d *Serious injuries includes injuries with a stand-down period of 21 or more days*.

Male dogs comprised slightly more of the race starts throughout the study period (*n* = 126,560/218,700; 57.87%) and New Zealand born dogs accounted for 166,923 (76.33%) of the race starts. The median race age for all starts was 31 months of age (IQR: 25–38 months) and during the study period dogs had a median of 28 racing starts (IQR: 13–51 starts). Of the fractures (*n* = 458), 286 (62.45%) occurred in male dogs and 307 (67.03%) were in New Zealand born dogs. The median race age at time of fracture was 33 months (IQR: 27–40 months) and the median race start number when the fracture occurred was 30.5 (IQR: 13–51). Of the lacerations (*n* = 641), 393 (61.31%) were in male dogs and 439 (68.49%) occurred in New Zealand born dogs. The median age for a reported laceration was 33 months (IQR: 26–40 months) and the median number of race starts for dogs that sustained a laceration was 29 (IQR: 13–54). Reflecting the underlying racing population, more soft-tissue injuries were reported for male dogs (*n* = 1,719/3,067; 56.05%) and for dogs of New Zealand origin (*n* = 2,287/3,067; 74.57%). The median race age for soft-tissue injuries was 33 months (IQR: 27–41 months) and the median race start number when a soft-tissue injury occurred was 30 (IQR: 13–55).

The univariable models for the outcomes of fracture, lacerations and soft-tissue injury are presented in Table 3 and full univariable results are provided in Supplementary Tables 1–3.

**Table 3:** Univariable logistic regression results of risk factors for fractures, lacerations, and soft-tissue injuries in racing greyhounds in New Zealand.

**Variable**		**Fracture**				**Laceration**				**Soft-tissue injury**		
		**OR^a^**	**95% CI**	***p*** **-value**		**OR^a^**	**95% CI**	***p*** **-value**		**OR^a^**	**95% CI**	***p-*** **value**
**Sex**												
Dog		Ref				Ref				Ref		
Bitch		0.83	0.68–1.00	0.05		0.87	0.74–1.02	0.08		1.08	1.00–1.16	0.04^*^
**Country of origin**												
New Zealand						Ref				Ref		
Australia		1.59	1.31–1.93	<0.001^*^		1.49	1.26–1.76	<0.001^*^		1.10	1.01–1.19	0.02^*^
**Race age (months)**												
14–25		Ref								Ref		
26–31		1.42	1.07–1.87	0.02^*^						1.17	1.05–1.30	<0.001^*^
32–38		1.76	1.33–2.31	<0.001^*^						1.42	1.27–1.58	<0.001^*^
39–77		1.79	1.36–2.35	<0.001^*^						1.80	1.63–1.99	<0.001^*^
**Career start number**												
1–13 starts						Ref						
14–28 starts						0.96	0.77–1.20	0.71				
29–51 starts						0.89	0.72–1.12	0.33				
51–231 starts						1.12	0.91–1.39	0.27				
**Days since previous race**												
<7		Ref				Ref				Ref		
7		1.17	0.94–1.47	0.16		0.89	0.74–1.07	0.20		0.90	0.83–0.99	0.02^*^
>7		1.40	1.12–1.74	<0.001^*^		0.93	0.77–1.12	0.42		1.11	1.02–1.21	0.01^*^
**Race type**												
Sprint				Ref		Ref				Ref		
Middle		0.91	0.75–1.11	0.36		0.94	0.79–1.11	0.44		0.89	0.82–0.96	<0.001^*^
Distance		1.24	0.68–2.26	0.49		1.47	0.91–2.35	0.11		0.81	0.61–1.07	0.14
**Race grade**												
Class 1		Ref				Ref				Ref		
Class 0		0.83	0.60–1.14	0.24		1.03	0.81–1.32	0.79		0.96	0.86–1.07	0.44
Class 2		1.06	0.80–1.41	0.67		1.16	0.93–1.46	0.19		1.10	0.99–1.22	0.07
Class 3		1.28	0.94–1.73	0.11		1.23	0.95–1.59	0.11		0.97	0.86–1.09	0.59
Class 4		1.41	1.02–1.96	0.04^*^		0.90	0.65–1.24	0.52		0.96	0.84–1.11	0.61
Class 5		1.80	1.36–2.39	<0.001^*^		1.43	1.11–1.85	0.01^*^		0.84	0.74–0.96	0.01^*^
Other		0.94	0.52–1.69	0.83		1.03	0.64–1.65	0.91		0.76	0.60–0.96	0.02^*^
**Racetrack**												
Track A		Ref				Ref				Ref		
Track B		0.60	0.36–0.99	0.05		0.81	0.54–1.21	0.30		0.74	0.57–0.96	0.02^*^
Track C		1.33	1.01–1.75	0.04^*^		1.64	1.30–2.07	<0.001^*^		2.61	2.29–2.97	<0.001^*^
Track D		0.99	0.77–1.26	0.92		1.19	0.96–1.47	0.11		3.36	3.01–3.75	<0.001^*^
Track E		0.82	0.57–1.18	0.28		1.06	0.79–1.43	0.70		1.68	1.43–1.97	<0.001^*^
Track F		0.72	0.46-1.14	0.17		0.54	0.34-0.86	0.01^*^		0.87	0.68-1.11	0.27
Track G		0.82	0.58–1.14	0.23		1.12	0.86–1.47	0.39		3.79	3.36–4.29	<0.001^*^
**Starting box**												
1		Ref				Ref				Ref		
2		1.51	1.00–2.29	0.05		1.06	0.76–1.46	0.74		1.05	0.91–1.21	0.48
3		1.69	1.12–2.53	0.01^*^		1.23	0.90–1.69	0.19		1.03	0.90–1.19	0.64
4		1.52	1.00–2.30	0.05		1.23	0.90–1.68	0.20		1.02	0.89–1.17	0.77
5		1.66	1.10–2.50	0.02^*^		1.16	0.85–1.60	0.35		0.97	0.84–1.11	0.63
6		1.57	1.04–2.38	0.03^*^		1.39	1.02–1.88	0.04^*^		0.98	0.85–1.13	0.80
7		1.52	1.00–2.30	0.05		1.06	0.76–1.46	0.74		0.89	0.77–1.03	0.12
8		1.95	1.31–2.90	<0.001^*^		0.93	0.67–1.30	0.68		0.87	0.75–1.01	0.07
**Season**												
Winter		Ref				Ref				Ref		
Spring		1.17	0.90–1.53	0.25		1.13	0.91–1.40	0.26		0.95	0.86–1.05	0.33
Summer		1.20	0.93–1.57	0.17		0.93	0.75–1.16	0.53		0.96	0.87–1.06	0.46
Autumn		1.34	1.04–1.74	0.03^*^		1.02	0.82–1.27	0.85		0.88	0.79–0.97	0.01^*^
**Race year**												
2018/2019		Ref				Ref				Ref		
2019/2020		0.84	0.63–1.13	0.25		1.19	0.94–1.52	0.16		1.19	1.08–1.32	<0.001^*^
2017/2018		0.83	0.63–1.11	0.21		0.97	0.75–1.24	0.78		0.72	0.65–0.81	<0.001^*^
2016/2017		0.92	0.69–1.22	0.56		0.90	0.70–1.17	0.44		0.63	0.55–0.71	<0.001^*^
2015/2016		0.99	0.74–1.34	0.97		1.21	0.94–1.56	0.15		0.89	0.79–1.00	0.05^*^
2014/2015		1.10	0.73–1.66	0.64		1.24	0.87–1.77	0.24		0.64	0.53–0.78	<0.001^*^

Statistically significant variables (p < 0.05) are marked with (*).

a *OR, Odds Ratio*.

### Fracture

The results for univariable screening of potential risk factors for fractures are presented in Table 3. The sex of the dog, country of origin, age at time of racing start, days since previous race start, race grade, racetrack, starting box position and season were selected for inclusion in the multivariable model. The final multivariable model for the outcome of fracture is presented in Table 4. No interaction terms were significant. After adjusting for the other variables in the model, the odds of fracture were higher in Australian dogs racing in New Zealand compared with New Zealand dogs. Greyhounds in the older age categories had a greater odds of fracture compared with the younger baseline group (14–25 months). The odds of fracture were higher for dogs that had not raced in the previous 7 days compared with those that had <7 days since their previous race. There were increased odds of fracture for dogs racing in class 5 compared to class 1 races. Increased odds of fracture were observed in dogs starting from all boxes compared to box 1, and if the dog started from box 8, the odds of fracture was nearly two times that of dogs starting from box 1 (OR: 1.95, 95% CI: 1.31-2.90).

**Table 4:** Multivariable logistic regression results of risk factors for fractures in racing greyhounds in New Zealand.

**Variable**	**Category**	**Coefficient**	**SE^a^**	**Adjusted OR^b^**	**95% CI**		***p-*** **value**
					**Lower**	**Upper**	
**Country of origin**							
	New Zealand	Ref					
	Australia	0.41	0.12	1.50	1.20	1.88	<0.001^*^
**Race age (months)**							
	14–25	Ref					
	26–31	0.23	0.15	1.26	0.94	1.70	0.12
	32–38	0.36	0.15	1.44	1.06	1.94	0.02^*^
	39–77	0.37	0.16	1.45	1.07	1.97	0.02^*^
**Days since previous race**							
	<7	Ref					
	7	0.13	0.12	1.14	0.90	1.44	0.28
	>7	0.29	0.12	1.33	1.06	1.67	0.01^*^
**Race grade**							
	Class 1	Ref					
	Class 0	−0.11	0.17	0.90	0.65	1.24	0.51
	Class 2	0.06	0.15	1.06	0.8	1.42	0.67
	Class 3	0.24	0.16	1.27	0.93	1.72	0.13
	Class 4	0.31	0.17	1.37	0.98	1.91	0.07
	Class 5	0.49	0.15	1.63	1.21	2.19	<0.001^*^
	Other	−0.01	0.30	0.99	0.55	1.80	0.97
**Starting box**							
	1	Ref					
	2	0.42	0.21	1.52	1.00	2.30	0.05
	3	0.52	0.21	1.68	1.12	2.53	0.01^*^
	4	0.42	0.21	1.52	1.00	2.31	0.05
	5	0.51	0.21	1.67	1.11	2.51	0.01^*^
	6	0.45	0.21	1.57	1.04	2.38	0.03^*^
	7	0.42	0.21	1.52	1.00	2.30	0.05
	8	0.67	0.20	1.95	1.31	2.90	<0.001^*^
**Season**							
	Winter	Ref					
	Spring	0.15	0.14	1.16	0.88	1.51	0.29
	Summer	0.18	0.14	1.20	0.92	1.57	0.18
	Autumn	0.28	0.13	1.33	1.03	1.72	0.03^*^

Statistically significant variables (p < 0.05) are marked with (*).

a *SE, Standard error*.

b *OR, Odds ratio*.

*4.7% of the variation in fractures, unexplained by fixed variables in the model, occurred at the trainer level*.

The fit of the model was assessed by residual values and the residual values had a median of 0.0026 (IQR: 0.0020–0.0034) and 0.0028 (IQR: 0.0021–0.0037) for starts without a fracture and those with a fracture occurring, respectively.

### Laceration

The results for univariable screening of potential risk factors for lacerations are presented in Table 3. The sex of the dog, country of origin, race type (distance), race grade, racetrack, starting box position and race year were selected for inclusion in the multivariable model. The final multivariable model for the outcome of laceration is presented in Table 5. No interaction terms were significant. After adjusting for the other variables in the model, the odds of laceration were higher in Australian dogs compared with the New Zealand dogs. The odds of laceration varied for each race grade compared with class 1. The odds of laceration increased at tracks C and D compared with track A, whereas the odds were decreased at track F compared with track A.

**Table 5:** Multivariable logistic regression results of risk factors for lacerations in racing greyhounds in New Zealand.

**Variable**	**Category**	**Coefficient**	**SE^a^**	**Adjusted OR^b^**	**95% CI**		***p*** **-value**
					**Lower**	**Upper**	
**Country of origin**							
	New Zealand	Ref					
	Australia	0.35	0.10	1.42	1.17	1.73	<0.001^*^
**Race grade**							
	Class 1	Ref					
	Class 0	0.10	0.13	1.10	0.86	1.41	0.45
	Class 2	0.14	0.12	1.15	0.91	1.45	0.24
	Class 3	0.19	0.13	1.21	0.93	1.56	0.15
	Class 4	−0.13	0.17	0.88	0.63	1.22	0.43
	Class 5	0.33	0.14	1.39	1.07	1.82	0.02^*^
	Other	0.10	0.24	1.10	0.69	1.78	0.68
**Racetrack**							
	Track A	Ref					
	Track B	−0.18	0.21	0.83	0.56	1.25	0.38
	Track C	0.54	0.15	1.72	1.27	2.32	<0.001^*^
	Track D	0.28	0.14	1.32	1.01	1.73	0.04^*^
	Track E	0.08	0.18	1.09	0.76	1.54	0.65
	Track F	−0.53	0.25	0.59	0.36	0.96	0.03^*^
	Track G	0.20	0.17	1.23	0.89	1.69	0.22

Statistically significant variables (p < 0.05) are marked with (*).

a *SE, Standard error*.

b *OR, Odds ratio*.

*5.4% of the variation in lacerations, unexplained by fixed variables in the model, occurred at the trainer level*.

The residual values had a median of 0.0026 (IQR: 0.0020–0.0034) and 0.0033 (IQR: 0.0025–0.0043) for starts without a laceration and those with a laceration occurring, respectively. The low residual values demonstrate that the model was a good fit.

### Soft-Tissue Injury

The results for univariable screening of potential risk factors for soft-tissue injury are presented in Table 3. The sex of the dog, country of origin, age at time of racing start, days since previous race start, race type (distance), race grade, race-track, starting box position, season and race year were selected for inclusion in the multivariable model. The multivariable model for the outcome of soft-tissue injuries is presented in Table 6. No interaction terms were significant. After accounting for the other variables in the model, the odds of soft-tissue injuries were increased for dogs aged between 39 and 77 months compared with those aged 14 and 25 months and the odds were decreased for dogs racing once a week compared to more than once a week. The odds of a soft-tissue injury occurring was increased for dogs in maiden races (class 0), compared with those in class 1. The odds of a soft-tissue injury varied for the different tracks; there was an increased odds of soft-tissue injury at track C, track D and track G compared with track A. There was variation in the odds of soft-tissue injury across the different racing years, in particular with the 2019/2020 season having an increased odds compared with the 2018/2019 racing season while the 2014/2015, 2016/2017, and 2017/2018 seasons all had a decreased odds compared to the 2018/2019 season.

**Table 6:** Multivariable logistic regression results of risk factors for soft-tissue injuries in racing greyhounds in New Zealand.

**Variable**	**Category**	**Coefficient**	**SE^a^**	**Adjusted OR^b^**	**95% CI**		***p*** **-value**
					**Lower**	**Upper**	
**Race age (months)**							
	14–25	Ref					
	26–31	0.62	0.06	1.06	0.95	1.19	0.29
	32–38	0.22	0.06	1.25	1.11	1.41	<0.001^*^
	39–77	0.44	0.06	1.55	1.37	1.75	<0.001^*^
**Days since previous race**							
	<7	Ref					
	7	−0.13	0.05	0.87	0.80	0.96	<0.001^*^
	>7	0.06	0.05	1.07	0.97	1.16	0.17
**Race distance type**							
	Sprint	Ref					
	Middle	−0.01	0.04	0.99	0.91	1.08	0.87
	Distance	−0.31	0.15	0.73	0.55	0.99	0.04^*^
**Race grade**							
	Class 1	Ref					
	Class 0	0.20	0.06	1.22	1.08	1.37	<0.001^*^
	Class 2	0.07	0.05	1.07	0.97	1.19	0.19
	Class 3	−0.28	0.06	0.97	0.86	1.10	0.67
	Class 4	−0.01	0.07	0.99	0.85	1.14	0.84
	Class 5	−0.14	0.07	0.87	0.76	1.01	0.07
	Other	−0.05	0.12	0.95	0.75	1.22	0.71
**Racetrack**							
	Track A	Ref					
	Track B	−0.27	0.14	0.76	0.58	1.00	0.05
	Track C	0.94	0.10	2.56	2.12	3.10	<0.001^*^
	Track D	1.23	0.08	3.44	2.94	4.02	<0.001^*^
	Track E	0.55	0.11	1.73	1.40	2.13	<0.001^*^
	Track F	−0.07	0.13	0.94	0.73	1.21	0.61
	Track G	1.46	0.09	4.31	3.65	5.10	<0.001^*^
**Season**							
	Winter	Ref					
	Spring	−0.09	0.05	0.91	0.82	1.01	0.01^*^
	Summer	−0.06	0.05	0.94	0.85	1.04	0.23
	Autumn	−0.11	0.05	0.90	0.81	0.99	0.03^*^
**Race year**							
	2018/2019	Ref					
	2019/2020	0.23	0.05	1.26	1.14	1.40	<0.001^*^
	2017/2018	−0.30	0.06	0.74	0.66	0.83	<0.001^*^
	2016/2017	−0.43	0.06	0.65	0.57	0.74	<0.001^*^
	2015/2016	−0.02	0.06	0.98	0.87	1.11	0.79
	2014/2015	−0.26	0.10	0.77	0.63	0.95	0.01^*^

Statistically significant variables (p < 0.05) are marked with (*).

a *SE, Standard Error*.

b *OR, Odds ratio*.

*14.2% of the variation in soft-tissue injuries, unexplained by fixed variables in the model, occurred at the trainer level*.

Goodness of fit was assessed by examining the residual values. The residual values had a median of 0.0026 (IQR: 0.0020–0.0034) and 0.0028 (IQR: 0.0023–0.0037) for starts without a soft-tissue injury and those with a soft-tissue injury occurring, respectively.

## Discussion

This study is the first to provide an assessment of the three most common injury categories that occur during racing: soft-tissue injuries, lacerations and fractures and has identified risk factors associated with these racing injuries in greyhounds in New Zealand. Injury types were analyzed separately as we hypothesized that the etiology and distribution of soft-tissue injuries, fractures and lacerations would differ greatly. The distribution of injuries in this study was similar to that described by Beer (6). These results were not unexpected, given that the structure and style of racing is similar in New Zealand and Australia. The most common injuries were reported as soft-tissue injuries, with most of these being classed as “moderate” severity (injuries requiring a stand-down period of 7–20 days). Although there were fewer fractures compared with lacerations and soft-tissue injuries, more than 95% were classed as either severe injuries (21 day stand down or more) or required euthanasia, suggesting a greater impact of a fracture on the future of the dogs racing career.

Greyhounds' that had a lower racing frequency had greater odds of fracture, which agrees with an earlier study examining injury risk and number of starts in the previous 60 days (7). However, historically there have been fewer opportunities for greyhounds to race (1, 7). In New Zealand, greyhounds have a consistent racing schedule with little variation in when dogs have a racing start. For this reason, investigating the association between time since previous racing start and risk of fracture provided information within a relevant timeframe. While we considered modeling in a similar manner to a previous study from New Zealand (number of racing starts in the previous 60 days) (7), the number of days since the previous racing start and the time-varying variables were collinear and days since the previous racing starts was nested within each of the time-varying variables. Therefore, investigating time between consecutive racing starts was considered more relevant for analyzing racing frequency because it was more likely to reflect the pathophysiology of the injury.

The accumulation of load and frequency of load sustained during training and racing exercise is implicated in the pathophysiology of bone injuries across a number of species; including racing greyhounds (14, 15). The nature of the resulting bone remodeling depends upon the magnitude and frequency of the load (3, 16, 17). Our results showed that the risk of fracture was associated with the greyhounds with the least racing-intensity exercise accumulated, as measured by more than 7 days between consecutive racing starts. This could be due to the requirement for bone to receive appropriate “specific” strain that reflects the load experienced during racing and high-intensity exercise (18–20). The appropriate load is a balance between the total volume of high-speed exercise, the rate at which this exercise is accumulated, and the recovery periods that allow adaptation of bones without reaching the fatigue life of the bone (21, 22). Analyzing the time period between consecutive racing starts provided temporal relevance indicating what had occurred around the time of the race. Increased risk of fracture in racing due to greater time between consecutive races was likely exacerbated by a survival bias (23), where the dogs that were not racing at least once a week were those that already had, or were prone to, health complications or had prior ailments. Previous injuries were not specifically investigated in this study, which may be a confounding factor for the association between fewer races in the previous 7 days and the risk of fracture.

The age of the greyhound at the time of the race start indirectly represented cumulative load, and our results found that older dogs had a greater odds of fracture compared to younger dogs. In New Zealand, greyhounds begin training and racing at a similar age (1, 2) and have a consistent training programme based around the opportunity to race at least once a week (2). Given this, older greyhounds had a higher risk of fracture compared to younger greyhounds due to the cumulative exposure to high-intensity load cycles. This result is in agreement with existing literature (6, 7), where increased age has been associated with racing injuries and serious tarsal injury in racing greyhounds.

Sicard et al. (9) previously reported that the injury rate in racing greyhounds increased as the grade of the race increased. In this study, dogs performing in a higher race grade (class five) were identified as having a greater risk of fracture compared with the class one dogs. Although race speed data were not provided, racing class is used an indirect measure of racing speed, as faster and more successful greyhounds would be racing in higher classes (9). Faster winning race speeds are associated with increasing injury risk (7, 9), and speed has been well-documented as a risk factor for musculoskeletal injury in racehorses (23–26). The association between speed and risk of fracture has been attributed to the increased forces and torque applied to the limbs with increased speed in racehorses (27). Over numerous load cycles the increased force results in increased microdamage (21, 28). It is hypothesized that the increased odds of fracture with Australian dogs reflects the higher racing grade (only high grade or high grade potential dogs are imported (1) and the previously described effect of increased strain at higher racing speeds as well as increased load cycles experienced by these higher grade dogs.

This study identified starting box number as a risk factor for fractures, which has not been previously reported. In New Zealand, the lure is on the inside rail, which is closest to starting box number one and furthest away from box number eight (which presented higher odds of fracture). The most likely hypothesis is a combination of the field of dogs galloping after the one lure, as well as the physical orientation and properties of the track. In conjunction, these alter the opportunities for greyhounds to obtain their desired path by the time they reach the first bend, which is also where the greyhounds are traveling the fastest (29, 30) and most injuries occur (9, 31–33). However, speed, track properties and the location of injuries on the track were not specifically investigated in this study, suggesting that further work is required to determine if the impact of starting box number can be modified to reduce future injuries.

We hypothesize that risk factors that were significantly associated with lacerations pertain primarily to environmental factors, as a greyhound must have come in contact with an object or another greyhound in order to sustain a laceration. For example, we hypothesize that many lacerations may occur at the end of the race when the greyhounds congregated at the lure where jostling and interference occur. At the end of a race, when the lure decelerated, the greyhounds were able to finish on the lure, resulting in the field of dogs within close proximity all competing for one target as well as the metal lure arm and rail on which the lure transverses. Recent studies have investigated path trajectories with different length lure arms (11) and it may be useful for future work to consider the effect of a larger or different style of lure to help mitigate some of these injuries. However, it should be noted that the location of the injury on the track was not accurately recorded in the dataset used in this study. Such data would be useful to allow more investigation of the locations that have higher risks of injury, which may enable modifications to made to reduce injury risk.

Soft-tissue injuries sustained by greyhounds during a race were predominantly muscle injuries with a relatively short stand down period imposed. There was a lack of specificity in how the soft-tissue injury data were reported and this limited precise description of the different muscle, tendon, and ligament injuries. However, the relatively short stand down periods (typically 1–20 days) assigned to the soft-tissue category of injuries suggest that most were muscular (not tendon or ligament). Such injuries could be considered a by-product of athletic pursuit, and the level to which they were detected demonstrates the sensitivity of veterinary examination to identifying soft-tissue injuries. It is hard to determine what soft-tissue injury was sustained as the information on the specific anatomical location of the injury was not provided in the database. There is a need for a more specific case definition of soft-tissue injuries to be described if potential risk-factors for muscle, tendon and ligament injuries are to be examined and misclassification bias is to be minimized.

The general trend for increasing odds of soft-tissue injury with increasing age seen in this study is consistent with findings from other racing animal models (34, 35). Additionally, the occurrence of musculoskeletal injuries is known to increase with age in military working dogs (36) and agility dogs (37). The association with age could be attributed to accumulated cyclic load and the changes in the regenerative potential of the affected tissues as the greyhound matures. A classic example of this interaction of cyclic load and changing tissue properties and regenerative potential is the loss of structural integrity of tendons associated with the aging process reported in thoroughbred racehorses (38, 39). Moreover, because the workload of racing greyhounds is consistent and highly repeatable, without consistent rest or spell periods (2), the risk of injury continues to increase, even though there is no increase in the weekly training volume.

The association between racetrack and injuries in greyhounds has been previously noted (9–11, 33, 40). Track related parameters, including the track radius, angle of banking and the track surface, predispose greyhounds to specific injuries (9, 10, 40, 41). However, this study found no clear pattern between tracks and odds of soft-tissue injuries in this study, despite the tracks having different geometries. Clustering at a regional level may be due to the different personnel (Stipendiary Stewards and on-track veterinarians) at different racetracks. Different Stipendiary Stewards are instructing different veterinarians to perform examinations across the different regions, potentially leading to variability in the level of reporting and inspection of greyhounds. For example, tracks D and G are located in the same region and despite the two tracks having different geometric features, they had a similar odds of soft-tissue injury compared to track A. This variation in soft-tissue injuries could, in part, be explained by the same personnel working at racetracks in similar areas, as well as the different personnel throughout New Zealand having a different interpretation on the level of precision and recording required for injury reporting. Given that descriptions of injuries were primarily based on clinical examination alone, there was potential that some reported injuries were misclassified. The lack of robust methods for injury detection and reporting, as well as potential misclassification of injuries could explain the differences in odds of soft-tissue injury at the difference race tracks.

The greyhound racing industry in New Zealand should look to moving toward a more detailed standardized injury reporting scheme which would provide more precise and descriptive details of the injuries sustained during racing. The broad description of the type and location of injuries needs to be improved to provide more robust data to describe the incidence of anatomically specific injuries so that the incidence and associated risk factors can be determined. The increase in soft-tissue injuries in the 2019/2020 racing season compared with the 2018/2019 season may be attributed to improvements in the intensity of injury surveillance and the efficiency of data capture having improved over time. As noted above, it is important to consider the potential bias in the detection, diagnosing and reporting of racing injuries, attributable to the methods of data collection and consistency of reporting (12).

## Conclusions

Soft-tissue injuries, lacerations and fractures were the three most common injuries of racing greyhounds in New Zealand in this study. The identification of these three injuries allowed specific risk factors to be analyzed for each outcome. Our findings demonstrated that fractures had a greater impact than soft-tissue injuries, however, fractures occurred at a lower frequency. To obtain a better understanding of the injuries associated with racing in New Zealand and in order to proactively manage racing greyhounds, efforts should be focused into improving the precision at which injuries are detected and reported. We found that dogs racing less frequently were more likely to sustain a fracture, the odds of fracture increased with increasing race speed and the odds of fracture were higher in older greyhounds compared with younger greyhounds. As in many forms of competitive sports, injuries are an inherent element of racing and in order to reduce the incidence rate of injuries, more thorough diagnostic tools are required for detection. To reduce the rate of injuries in racing greyhounds, efforts should be focused into investigating and screening those greyhounds who have not raced frequently. Early detection of injuries may help to identify dogs that are at risk of sustaining a racing injury. This study provides a useful benchmark from which to assess the effectiveness of future strategies implemented by the industry.

## Data Availability Statement

The data analyzed in this study is subject to the following licenses/restrictions: The data analyzed in this study was obtained from, and is owned by, Greyhound Racing New Zealand. Requests to access these datasets should be directed to Greyhound Racing New Zealand, greyhound@grnz.co.nz.

## Ethics Statement

Ethical review and approval was not required for this study because the research utilized retrospective industry data on race start information which was collected from a database.

## Author Contributions

AP was responsible for conception and design of the work, the acquisition, analysis, and interpretation of data, drafting and revising, and gave final approval for it to be published. CB and CR assisted in the conception and design of the work, the acquisition, analysis, and interpretation of data, drafting and revising, and gave final approval for it to be published. KS was responsible for conception of the study, drafting and revising, and gave final approval for it to be published. AG assisted with the drafting and revising of the manuscript before giving approval for it to be published. All authors agree to be accountable for all aspects of the work in ensuring that questions related to the accuracy or integrity of any part of the work are appropriately investigated and resolved.

## Funding

This study received funding from Greyhound Racing New Zealand. The funder was not involved in the study design, collection, analysis, interpretation of data, nor the writing of this article or the decision to submit it for publication.

## Conflict of Interest

The current study uses historical data from the 2014–2020 racing seasons. Greyhound Racing New Zealand provided the spreadsheet from a database that is managed by an external statistics company. While the funders provided access to the database, they had no role in the design of the study, analysis of the data, interpretation of the data, writing of the manuscript, or in the decision to publish the results. The authors declare that the research was conducted in the absence of any commercial or financial relationships that could be construed as a potential conflict of interest.

## Publisher's Note

All claims expressed in this article are solely those of the authors and do not necessarily represent those of their affiliated organizations, or those of the publisher, the editors and the reviewers. Any product that may be evaluated in this article, or claim that may be made by its manufacturer, is not guaranteed or endorsed by the publisher.
