# Supplementary material for: Risk-Factors for Soft-Tissue Injuries, Lacerations and Fractures During Racing in Greyhounds in New Zealand
Source: Front Vet Sci. 2021 Dec 3;8:737146. doi: 10.3389/fvets.2021.737146 (PMC8678076; doi:10.3389/fvets.2021.737146)
Supplement: Supplementary file 1 [file Table_1.DOCX]

**Supplementary Table 1**

Results of univariable logistic regression screening of variables associated with fractures in racing greyhounds in New Zealand.

| Variable | Category | Coefficient | SE^a^ | Unadjusted OR^b^ | 95% CI | | p-value^c^ | LRS^d^ p-value |
| --- | --- | --- | --- | --- | --- | --- | --- | --- |
|  |  |  |  |  | Lower | Upper |  |  |
| Sex |  |  |  |  |  |  |  | 0.05 |
|  | Dog | Ref |  |  |  |  |  |  |
|  | Bitch | -0.19 | 0.10 | 0.83 | 0.68 | 1.00 | 0.05 |  |
| Country of Origin |  |  |  |  |  |  |  | 0.00 |
|  | New Zealand | Ref |  |  |  |  |  |  |
|  | Australia | 0.46 | 0.10 | 1.59 | 1.31 | 1.93 | 0.00 |  |
| Race Age (months) |  |  |  |  |  |  |  | 0.00 |
|  | 14-25 | Ref |  |  |  |  |  |  |
|  | 26-31 | 0.35 | 0.14 | 1.42 | 1.07 | 1.87 | 0.02 |  |
|  | 32-38 | 0.56 | 0.14 | 1.76 | 1.33 | 2.31 | 0.00 |  |
|  | 39-77 | 0.58 | 0.14 | 1.79 | 1.36 | 2.35 | 0.00 |  |
| Days since Previous Race |  |  |  |  |  |  |  | 0.01 |
|  | <7 | Ref |  |  |  |  |  |  |
|  | 7 | 0.16 | 0.12 | 1.17 | 0.94 | 1.47 | 0.16 |  |
|  | >7 | 0.33 | 0.11 | 1.40 | 1.12 | 1.74 | 0.00 |  |
| Race Type |  |  |  |  |  |  |  | 0.49 |
|  | Sprint | Ref |  |  |  |  |  |  |
|  | Middle | -0.09 | 0.10 | 0.91 | 0.75 | 1.11 | 0.36 |  |
|  | Distance | 0.21 | 0.31 | 1.24 | 0.68 | 2.26 | 0.49 |  |
| Race Grade |  |  |  |  |  |  |  | 0.00 |
|  | Class 1 | Ref |  |  |  |  |  |  |
|  | Class 0 | -0.19 | 0.16 | 0.83 | 0.60 | 1.14 | 0.24 |  |
|  | Class 2 | 0.06 | 0.14 | 1.06 | 0.80 | 1.41 | 0.67 |  |
|  | Class 3 | 0.24 | 0.15 | 1.28 | 0.94 | 1.73 | 0.11 |  |
|  | Class 4 | 0.35 | 0.17 | 1.41 | 1.02 | 1.96 | 0.04 |  |
|  | Class 5 | 0.59 | 0.14 | 1.80 | 1.36 | 2.39 | 0.00 |  |
|  | Other | -0.06 | 0.30 | 0.94 | 0.52 | 1.69 | 0.83 |  |
| Racetrack |  |  |  |  |  |  |  | 0.01 |
|  | Track A | Ref |  |  |  |  |  |  |
|  | Track B | -0.52 | 0.26 | 0.60 | 0.36 | 0.99 | 0.05 |  |
|  | Track C | 0.28 | 0.14 | 1.33 | 1.01 | 1.75 | 0.04 |  |
|  | Track D | -0.01 | 0.13 | 0.99 | 0.77 | 1.26 | 0.92 |  |
|  | Track E | -0.20 | 0.18 | 0.82 | 0.57 | 1.18 | 0.28 |  |
|  | Track F | -0.32 | 0.23 | 0.72 | 0.46 | 1.14 | 0.17 |  |
|  | Track G | -0.20 | 0.17 | 0.82 | 0.58 | 1.14 | 0.23 |  |
| Starting Box |  |  |  |  |  |  |  | 0.08 |
|  | 1 | Ref |  |  |  |  |  |  |
|  | 2 | 0.41 | 0.21 | 1.51 | 1.00 | 2.29 | 0.05 |  |
|  | 3 | 0.52 | 0.21 | 1.69 | 1.12 | 2.53 | 0.01 |  |
|  | 4 | 0.42 | 0.21 | 1.52 | 1.00 | 2.30 | 0.05 |  |
|  | 5 | 0.51 | 0.21 | 1.66 | 1.10 | 2.50 | 0.02 |  |
|  | 6 | 0.45 | 0.21 | 1.57 | 1.04 | 2.38 | 0.03 |  |
|  | 7 | 0.42 | 0.21 | 1.52 | 1.00 | 2.30 | 0.05 |  |
|  | 8 | 0.67 | 0.20 | 1.95 | 1.31 | 2.90 | 0.00 |  |
| Season |  |  |  |  |  |  |  | 0.16 |
|  | Winter | Ref |  |  |  |  |  |  |
|  | Spring | 0.16 | 0.14 | 1.17 | 0.90 | 1.53 | 0.25 |  |
|  | Summer | 0.19 | 0.13 | 1.20 | 0.93 | 1.57 | 0.17 |  |
|  | Autumn | 0.29 | 0.13 | 1.34 | 1.04 | 1.74 | 0.03 |  |
| Race Year |  |  |  |  |  |  |  | 0.61 |
|  | 2018/2019 | Ref |  |  |  |  |  |  |
|  | 2019/2020 | -0.17 | 0.15 | 0.84 | 0.63 | 1.13 | 0.25 |  |
|  | 2017/2018 | -0.18 | 0.15 | 0.83 | 0.63 | 1.11 | 0.21 |  |
|  | 2016/2017 | -0.08 | 0.15 | 0.92 | 0.69 | 1.22 | 0.56 |  |
|  | 2015/2016 | -0.01 | 0.15 | 0.99 | 0.74 | 1.34 | 0.97 |  |
|  | 2014/2015 | 0.10 | 0.21 | 1.10 | 0.73 | 1.66 | 0.64 |  |
| ^a^ SE: Standard Error | | | | | | | | |
| ^b^ OR: Odds Ratio | | | | | | | | |
| ^c^ Wald p-value | | | | | | | | |
| ^d^ LRS p-value: Likelihood ratio statistic p-value | | | | | | | | |
