# Supplementary material for: Risk-Factors for Soft-Tissue Injuries, Lacerations and Fractures During Racing in Greyhounds in New Zealand
Source: Front Vet Sci. 2021 Dec 3;8:737146. doi: 10.3389/fvets.2021.737146 (PMC8678076; doi:10.3389/fvets.2021.737146)
Supplement: Supplementary file 2 [file Table_2.DOCX]

**Supplementary Table 2**

Results of univariable logistic regression screening of variables associated with laceration injuries in racing greyhounds in New Zealand.

| Variable | Category | Coefficient | SE^a^ | Unadjusted OR^b^ | 95% CI | | p-value^c^ | LRS^d^ p-value |
| --- | --- | --- | --- | --- | --- | --- | --- | --- |
|  |  |  |  |  | Lower | Upper |  |  |
| Sex |  |  |  |  |  |  |  | 0.08 |
|  | Dog | Ref |  |  |  |  |  |  |
|  | Bitch | -0.14 | 0.08 | 0.87 | 0.74 | 1.02 | 0.08 |  |
| Country of Origin |  |  |  |  |  |  |  |  |
|  | New Zealand | Ref |  |  |  |  |  | 0.00 |
|  | Australia | 0.40 | 0.09 | 1.49 | 1.26 | 1.76 | 0.00 |  |
| Career Start Number |  |  |  |  |  |  |  | 0.22 |
|  | 1-13 | Ref |  |  |  |  |  |  |
|  | 14-28 | -0.04 | 0.11 | 0.96 | 0.77 | 1.20 | 0.71 |  |
|  | 29-51 | -0.11 | 0.11 | 0.89 | 0.72 | 1.12 | 0.33 |  |
|  | 52-231 | 0.12 | 0.11 | 1.12 | 0.91 | 1.39 | 0.27 |  |
| Days since Previous Race |  |  |  |  |  |  |  | 0.43 |
|  | <7 | Ref |  |  |  |  |  |  |
|  | 7 | -0.12 | 0.09 | 0.89 | 0.74 | 1.07 | 0.20 |  |
|  | >7 | -0.08 | 0.10 | 0.93 | 0.77 | 1.12 | 0.42 |  |
| Race Type |  |  |  |  |  |  |  | 0.21 |
|  | Sprint | Ref |  |  |  |  |  |  |
|  | Middle | -0.07 | 0.09 | 0.94 | 0.79 | 1.11 | 0.44 |  |
|  | Distance | 0.38 | 0.24 | 1.47 | 0.91 | 2.35 | 0.11 |  |
| Race Grade |  |  |  |  |  |  |  | 0.08 |
|  | Class 1 | Ref |  |  |  |  |  |  |
|  | Class 0 | 0.03 | 0.13 | 1.03 | 0.81 | 1.32 | 0.79 |  |
|  | Class 2 | 0.15 | 0.12 | 1.16 | 0.93 | 1.46 | 0.19 |  |
|  | Class 3 | 0.21 | 0.13 | 1.23 | 0.95 | 1.59 | 0.11 |  |
|  | Class 4 | -0.11 | 0.17 | 0.90 | 0.65 | 1.24 | 0.52 |  |
|  | Class 5 | 0.36 | 0.13 | 1.43 | 1.11 | 1.85 | 0.01 |  |
|  | Other | 0.03 | 0.24 | 1.03 | 0.64 | 1.65 | 0.91 |  |
| Racetrack |  |  |  |  |  |  |  | 0.00 |
|  | Track A | Ref |  |  |  |  |  |  |
|  | Track B | -0.21 | 0.20 | 0.81 | 0.54 | 1.21 | 0.30 |  |
|  | Track C | 0.49 | 0.12 | 1.64 | 1.30 | 2.07 | 0.00 |  |
|  | Track D | 0.17 | 0.11 | 1.19 | 0.96 | 1.47 | 0.11 |  |
|  | Track E | 0.06 | 0.15 | 1.06 | 0.79 | 1.43 | 0.70 |  |
|  | Track F | -0.62 | 0.24 | 0.54 | 0.34 | 0.86 | 0.01 |  |
|  | Track G | 0.12 | 0.14 | 1.12 | 0.86 | 1.47 | 0.39 |  |
| Starting Box |  |  |  |  |  |  |  | 0.22 |
|  | 1 | Ref |  |  |  |  |  |  |
|  | 2 | 0.05 | 0.17 | 1.06 | 0.76 | 1.46 | 0.74 |  |
|  | 3 | 0.21 | 0.16 | 1.23 | 0.90 | 1.69 | 0.19 |  |
|  | 4 | 0.20 | 0.16 | 1.23 | 0.90 | 1.68 | 0.20 |  |
|  | 5 | 0.15 | 0.16 | 1.16 | 0.85 | 1.60 | 0.35 |  |
|  | 6 | 0.33 | 0.16 | 1.39 | 1.02 | 1.88 | 0.04 |  |
|  | 7 | 0.06 | 0.17 | 1.06 | 0.76 | 1.46 | 0.74 |  |
|  | 8 | -0.07 | 0.17 | 0.93 | 0.67 | 1.30 | 0.68 |  |
| Season |  |  |  |  |  |  |  | 0.39 |
|  | Winter | Ref |  |  |  |  |  |  |
|  | Spring | 0.12 | 0.11 | 1.13 | 0.91 | 1.40 | 0.26 |  |
|  | Summer | -0.07 | 0.11 | 0.93 | 0.75 | 1.16 | 0.53 |  |
|  | Autumn | 0.02 | 0.11 | 1.02 | 0.82 | 1.27 | 0.85 |  |
| Race Year |  |  |  |  |  |  |  | 0.12 |
|  | 2018/2019 | Ref |  |  |  |  |  |  |
|  | 2019/2020 | 0.18 | 0.12 | 1.19 | 0.94 | 1.52 | 0.16 |  |
|  | 2017/2018 | -0.04 | 0.13 | 0.97 | 0.75 | 1.24 | 0.78 |  |
|  | 2016/2017 | -0.10 | 0.13 | 0.90 | 0.70 | 1.17 | 0.44 |  |
|  | 2015/2016 | 0.19 | 0.13 | 1.21 | 0.94 | 1.56 | 0.15 |  |
|  | 2014/2015 | 0.21 | 0.18 | 1.24 | 0.87 | 1.77 | 0.24 |  |
| ^a^ SE: Standard Error | | | | | | | | |
| ^b^ OR: Odds Ratio | | | | | | | | |
| ^c^ Wald p-value | | | | | | | | |
| ^d^ LRS p-value: Likelihood ratio statistic p-value | | | | | | | | |
