# Supplementary material for: Risk-Factors for Soft-Tissue Injuries, Lacerations and Fractures During Racing in Greyhounds in New Zealand
Source: Front Vet Sci. 2021 Dec 3;8:737146. doi: 10.3389/fvets.2021.737146 (PMC8678076; doi:10.3389/fvets.2021.737146)
Supplement: Supplementary file 3 [file Table_3.pdf]

Supplementary Table 3

Results of univariable logistic regression screening of variables associated with soft-tissue injuries in racing greyhounds in New Zealand.

Variable	Category	Coefficient	SE[a]	Unadjusted OR[b]	95% CI		p-value[c]	LRS[d] p-value
					Lower	Upper		
Sex								0.04
	Dog	Ref						
	Bitch	0.08	0.04	1.08	1.00	1.16	0.04	
Country of Origin								0.02
	New Zealand	Ref						
	Australia	0.10	0.04	1.10	1.01	1.19	0.02	
Race Age (months)								0.00
	14-25	Ref						
	26-31	0.16	0.05	1.17	1.05	1.30	0.00	
	32-38	0.35	0.05	1.42	1.27	1.58	0.00	
	39-77	0.59	0.05	1.80	1.63	1.99	0.00	
Days since Previous Race								0.00
	<7	Ref						
	7	-0.10	0.04	0.90	0.83	0.99	0.02	
	>7	0.11	0.04	1.11	1.02	1.21	0.01	
Race Type								0.00
	Sprint	Ref						
	Middle	-0.12	0.04	0.89	0.82	0.96	0.00	
	Distance	-0.21	0.15	0.81	0.61	1.07	0.14	
Race Grade								0.00
	Class 1	Ref						
	Class 0	-0.04	0.06	0.96	0.86	1.07	0.44	
	Class 2	0.09	0.05	1.10	0.99	1.22	0.07	

	Class 3	-0.03	0.06	0.97	0.86	1.09	0.59	
	Class 4	-0.04	0.07	0.96	0.84	1.11	0.61	
	Class 5	-0.17	0.07	0.84	0.74	0.96	0.01	
	Other	-0.28	0.12	0.76	0.60	0.96	0.02	
Racetrack								0.00
	Track A	Ref						
	Track B	-0.30	0.13	0.74	0.57	0.96	0.02	
	Track C	0.96	0.07	2.61	2.29	2.97	0.00	
	Track D	1.21	0.06	3.36	3.01	3.75	0.00	
	Track E	0.52	0.08	1.68	1.43	1.97	0.00	
	Track F	-0.14	0.12	0.87	0.68	1.11	0.27	
	Track G	1.33	0.06	3.79	3.36	4.29	0.00	
Starting Box								0.11
	1	Ref						
	2	0.05	0.07	1.05	0.91	1.21	0.48	
	3	0.03	0.07	1.03	0.90	1.19	0.64	
	4	0.02	0.07	1.02	0.89	1.17	0.77	
	5	-0.04	0.07	0.97	0.84	1.11	0.63	
	6	-0.02	0.07	0.98	0.85	1.13	0.80	
	7	-0.11	0.07	0.89	0.77	1.03	0.12	
	8	-0.14	0.07	0.87	0.75	1.01	0.07	
Season								
	Winter	Ref						0.08
	Spring	-0.05	0.05	0.95	0.86	1.05	0.33	
	Summer	-0.04	0.05	0.96	0.87	1.06	0.46	
	Autumn	-0.13	0.05	0.88	0.79	0.97	0.01	
Race Year								0.00
	2018/2019	Ref						
	2019/2020	0.18	0.05	1.19	1.08	1.32	0.00	
	2017/2018	-0.32	0.06	0.72	0.65	0.81	0.00	
	2016/2017	-0.47	0.06	0.63	0.55	0.71	0.00	

	2015/2016	-0.12	0.06	0.89	0.79	1.00	0.05
	2014/2015	-0.44	0.10	0.64	0.53	0.78	0.00

[a] SE: Standard Error

[b] OR: Odds Ratio

[c] Wald p-value

[d] LRS p-value: Likelihood ratio statistic p-value
