# Supplementary material for: Risk-Factors for Soft-Tissue Injuries, Lacerations and Fractures During Racing in Greyhounds in New Zealand
Source: Front Vet Sci. 2021 Dec 3;8:737146. doi: 10.3389/fvets.2021.737146 (PMC8678076; doi:10.3389/fvets.2021.737146)
Supplement: Supplementary file 4 [file Image_1.DOCX]

Supplementary Material

**
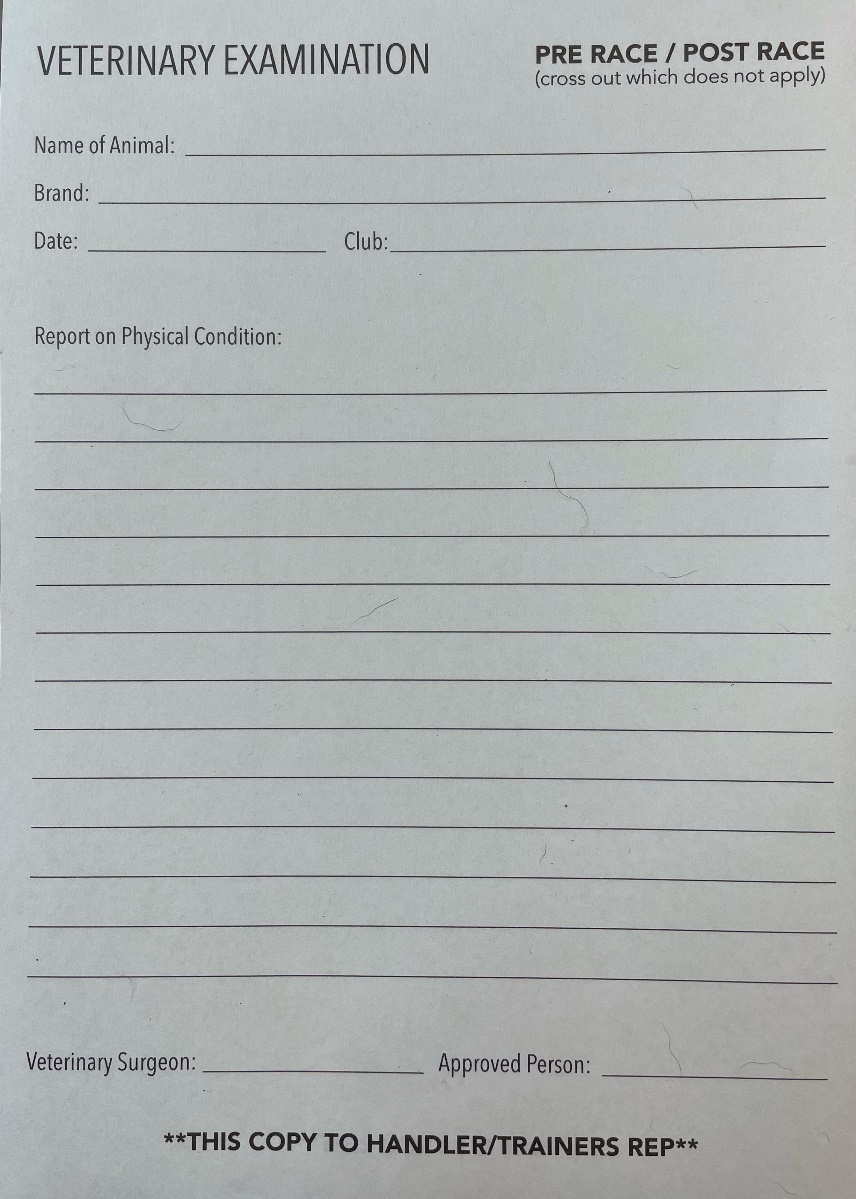
**

**Supplementary Figure 1.** Standardized injury reporting form used by on-track veterinarians for recording details of injuries sustained at the racetrack.
